# The host range restriction of bat-associated no-known-vector flaviviruses occurs post-entry

**DOI:** 10.1099/jgv.0.001647

**Published:** 2021-09-06

**Authors:** Jermilia Charles, Chandra S. Tangudu, Daniel Nunez-Avellaneda, Aaron C. Brault, Bradley J. Blitvich

**Affiliations:** ^1^​ Department of Veterinary Microbiology and Preventive Medicine, College of Veterinary Medicine, Iowa State University, Ames, Iowa, USA; ^2^​ Division of Vector-Borne Diseases, Centers for Disease Control and Prevention, Fort Collins, Colorado, USA

**Keywords:** chimeric, entry, flavivirus, host range, no-known-vector flavivirus, Rio Bravo virus

## Abstract

Most flaviviruses are transmitted horizontally between vertebrate hosts by haematophagous arthropods. Others exhibit host ranges restricted to vertebrates or arthropods. Vertebrate-specific flaviviruses are commonly referred to as no-known-vector (NKV) flaviviruses and can be separated into bat- and rodent-associated NKV flaviviruses. Rio Bravo virus (RBV) is one of eight recognized bat-associated NKV (B-NKV) flaviviruses. Studies designed to identify the genetic determinants that condition the host range restriction of B-NKV flaviviruses have never been performed. To investigate whether the host range restriction occurs at the level of attachment or entry, chimeric flaviviruses were created by inserting the pre-membrane and envelope protein genes of RBV into the genetic backbones of yellow fever virus (YFV) and Zika virus (ZIKV), two mosquito-borne flaviviruses associated with human disease. The chimeric viruses infected both vertebrate and mosquito cells. In vertebrate cells, all viruses produced similar mean peak titres, but the chimeric viruses grew more slowly than their parental viruses during early infection. In mosquito cells, the chimeric virus of YFV and RBV grew more slowly than YFV at early post-inoculation time points, but reached a similar mean peak titre. In contrast, the chimeric virus of ZIKV and RBV produced a mean peak titre that was approximately 10-fold lower than ZIKV. The chimeric virus of YFV and RBV produced an intermediate plaque phenotype, while the chimeric virus of ZIKV and RBV produced smaller plaques than both parental viruses. To conclude, we provide evidence that the structural glycoproteins of RBV permit entry into both mosquito and vertebrate cells, indicating that the host range restriction of B-NKV flaviviruses is mediated by a post-attachment/entry event.

## Introduction

All viruses in the genus *Flavivirus* (family *Flaviviridae*) possess a single-stranded, positive-sense RNA genome of 9.2–11 kb that encodes a 5′ untranslated region (UTR), a long open reading frame and a 3′ UTR [1, 2]. The open reading frame encodes a large polyprotein that is co- and post-translationally cleaved by viral and host proteases to generate three structural proteins, designated the capsid (C), pre-membrane/membrane (prM/M) and envelope (E) proteins, and at least seven nonstructural (NS) proteins in the gene order: 5′-C-prM(M)-E-NS1-NS2A-NS2B-NS3-NS4A-2K-NS4B-NS5-3′. The E protein binds to host cell attachment factors and receptors during attachment and entry [3]. The prM protein protects the E protein from undergoing an irreversible conformational change as the virion is secreted through acidified sorting compartments and is then cleaved into a soluble pr peptide and virion-associated M protein [4]. The E and prM proteins, unlike the C protein, are glycosylated, and both protrude from the host-derived lipid membrane that surrounds the capsid of the mature virion [5].

Despite a common genomic organization, flaviviruses possess fundamental differences in their natural host ranges and transmission cycles [1, 6, 7]. Most flaviviruses cycle between haematophagous arthropods and vertebrate hosts and can be further divided into mosquito- and tick-borne flaviviruses. Yellow fever virus (YFV) and Zika virus (ZIKV) are two examples of mosquito-borne flaviviruses (MBFVs) [8, 9]. Other flaviviruses have restricted host ranges, replicating only in vertebrates or insects [6, 7, 10, 11]. Vertebrate-specific flaviviruses are usually referred to as no-known-vector (NKV) flaviviruses and can be separated into bat- and rodent-associated NKV flaviviruses. The best-characterized bat- and rodent-associated NKV flavivirus viruses are Rio Bravo virus (RBV) and Modoc virus (MODV), respectively.

Eight bat-associated NKV (B-NKV) flaviviruses are recognized by The International Committee on Taxonomy of Viruses: Bukalasa bat virus (BBV), Carey Island virus (CIV), Dakar bat virus (DBV), Entebbe bat virus (ENTV), Montana myotis leukoencephalitis virus (MMLV), Phnom Penh bat virus (PPBV), RBV and Yokose virus (YOKV) [1]. Batu Cave virus (BCV) is a subtype of PPBV and Sokoluk virus (SOKV) is a subtype of ENTV. B-NKV flaviviruses can be further separated into the RBV group (BBV, BCV, CIV, DBV, MMLV, PPBV and RBV) and the Entebbe bat virus group (ENTV, SOKV and YOKV). Evidence is accumulating that ENTV group viruses replicate in arthropod cells and therefore should not be classified as NKV flaviviruses [12–14]. There is no evidence to suggest that RBV group viruses replicate in arthropod cells.

RBV is the prototype B-NKV flavivirus [15]. All isolations of RBV in nature have been made exclusively from bats, which appear to become persistently infected [15–19]. The virus has been isolated from the salivary glands and saliva of naturally infected bats, indicating that horizontal transmission could occur through aerosol exposure or salivary contact. RBV replicates in bat lung cells, in addition to monkey, rodent and human cells, and primary chick embryo fibroblasts, but cannot replicate in *Aedes albopictus*, *Aedes dorsalis* or *Culex tarsalis* mosquito cell lines [14, 20–23]. Studies designed to identify the genetic elements that condition the host restriction of RBV or any other B-NKV flavivirus have never been performed. The goal of this study was to determine whether this host range restriction occurs at virus attachment or entry.

## Methods

### Cell lines

The cell lines used in this study are as follows: *Aedes albopictus* (C6/36), African green monkey kidney (Vero), *Anopheles gambiae* (Sua 4.0), baby hamster kidney (BHK-21), *Culex tarsalis* (CT), duck embryonic fibroblast (DEF), murine microglia, rhesus macaque (LLC-MK2), *Spodoptera frugiperda* (Sf9) and *Trichoplusia ni* (BTI-Tn-5B1-4) cells. Some cell lines were obtained from the American Type Culture Collection (C6/36, Vero, BHK-21, DEF, LLC-MK2 and Sf9 cells), BEI Resources (Sua 4.0 cells) and Thermo Fisher Scientific (BTI-Tn-5B1-4 cells). Others were generously provided by Dr Gregory Ebel at Colorado State University in Fort Collins, Colorado, USA (CT cells) and Dr Anumantha Kanthasamy at Iowa State University in Ames, Iowa, USA (murine microglia cells). The microglia cells were originally generated by Dr Douglas Golenbock at University of Massachusetts Medical School in Worcester, Massachusetts, USA [24]. Cells were cultured in Dulbecco’s modified Eagle’s medium (all mammalian cells), Eagle’s minimum essential medium (DEF cells), Express Five medium (BTI-Tn-5B1-4 cells), Liebovitz L15 medium (C6/36 cells), Schneider’s Drosophila medium (CT and Sua 4.0 cells) and SF-900 medium (Sf9 cells) (all cell culture media from Thermo Fisher Scientific). All media were supplemented with 10 % foetal bovine serum (FBS), 2 mM l-glutamine, 100 units ml^−1^ of penicillin and 100 µg ml^−1^ of streptomycin, with the exception of the Express Five and SF-900 media, which were serum-free. Vertebrate cells were cultured at 37 °C with 5 % CO_2_ and invertebrate cells were cultured at 28 °C.

### Viruses

RBV (strain M64) and YFV (strain YF-17D) were obtained from the virus reference collections maintained at the University of Texas Medical Branch in Galveston, Texas, USA. ZIKV (strain PRVABC59) was obtained from the World Health Organization Center for Arbovirus Reference and Research maintained at the Division of Vector-Borne Infectious Diseases, Centers for Disease Control and Prevention in Fort Collins, Colorado, USA. Viruses underwent no more than three additional passages in cell culture in our laboratories.

### Plaque assays

To measure viral titres, viruses were subjected to serial 10-fold dilutions, inoculated onto confluent monolayers of Vero cells in 35 mm culture dishes and incubated at 37 °C for 60 min. Three millilitres of neutral red-deficient minimum essential medium (Thermo Fisher Scientific) supplemented with 2 % FBS, antibiotics and 1 % agar was added to each well. Plates were incubated at 37 °C for 5 days (chimeric viruses, RBV and ZIKV) or 6 days (YFV). Another 3 ml of the same medium containing 0.22 % neutral red was then added to each well, and plaques were counted 24 h later. Viral titres were expressed as plaque-forming units (p.f.u.) ml^−1^.

### Production of chimeras

**Fig. 1.**
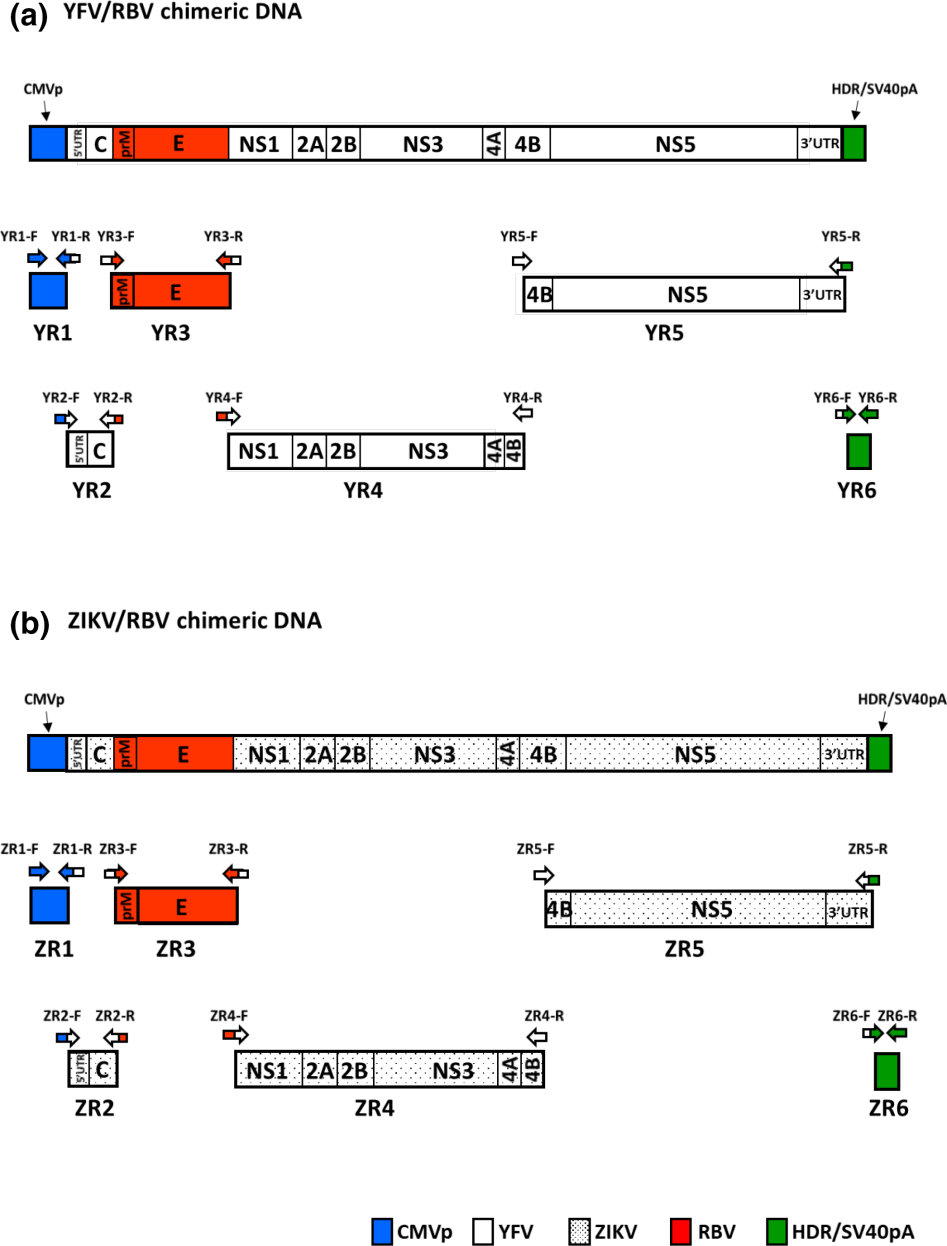
A schematic of the strategy used to generate the full-length chimeric infectious cDNAs of (a) YFV and RBV and (b) ZIKV and RBV. Genetic regions are colour-coded: cytomegalovirus promoter (CVMp), blue; hepatitis delta virus anti-genomic ribozyme and simian virus 40 polyadenylation signal (HDR/SV40pA) sequences, green; RBV sequence, red; YFV sequence, white; and ZIKV sequence, white with black dots. Primers are indicated by arrows.

Viral chimeras were created by inserting the prM-E genes of RBV into the genetic backbones of YFV and ZIKV. Each chimera was generated from six overlapping PCR products that were joined together by Gibson assembly (Fig. 1). PCRs were performed using the primers listed in Tables 1 and 2. Primers were designed such that each fragment contained a 15–26 bp overlap with the adjacent fragment(s). In some cases, this required the use of chimeric primers (i.e. half YFV or ZIKV sequence and half RBV sequence) that acted as linkers to fuse reaction products. Assembly reactions were performed such that there was the human cytomegalovirus (CMV) promoter sequence upstream of the chimeric flavivirus DNAs and the hepatitis delta virus anti-genomic ribozyme (HDR) sequence followed by the simian virus 40 polyadenylation signal (SV40pA) sequence downstream of the chimeric flavivirus DNAs. The CMV promoter, HDR and SV40pA were amplified from plasmids generously provided by Xavier de Lamballerie at Aix Marseille Universite in Marseille, France [25].

**Table 1.** Primers used during the construction of full-length YFV/RBV chimeric DNAs

Primer	Polarity	Sequence*	Genomic location†
YR1-F	Sense	5′ GAATAAGGGCGACACGGAAATGTC 3′	–
YR1-R	Antisense	5′ CACACAGGATTTACTCGGTTCACTAAACGAG 3′	1–13
YR2-F	Sense	5′ GTTTAGTGAACCGAGTAAATCCTGTGTGC 3′	1–16
YR2-R	Antisense	5′ CATAGCCATCAATCCACCCGTCATCAACAGC 3′	463–493
YR3-F	Sense	5′ CGGGTGGATTGATGGCTATGCAGGTATCCC 3′	474–503
YR3-R	Antisense	5′ GCATCCTTGATCTCCCATGACTCCAGTTGTAAATC 3′	2406–2440
YR4-F	Sense	5′ GAGTCATGGGAGATCAAGGATGCGCCATCAACTTTGG 3′	2418–2454
YR4-R	Antisense	5′ CTGTTATTGAATTCCAGCCACTGACC 3′	7153–7178
YR5-F	Sense	5′ GGTCAGTGGCTGGAATTCAATAACAG 3′	7153–7178
YR5-R	Antisense	5′ TGCCGGCCAGAGGTTTTGTGT 3′	10,829–10,838
YR6-F	Sense	5′ CAAAACCTCTAGCCGGCATGGTC 3′	10,826–10,838
YR6-R	Antisense	5′ GCTGCGCAACTGTTGGGAAG 3′	–

*Sequences are colour-coded: cytomegalovirus promoter, blue; yellow fever virus, black; Rio Bravo virus, red; hepatitis delta virus anti-genomic ribozyme sequence and simian virus 40 polyadenylation signal, green

†Location of the primer binding sites in the chimeric flavivirus genome with numbers starting at the first nucleotide of the flavivirus 5′ untranslated region (not the first nucleotide of the promoter sequence).

**Table 2.** Primers used during the construction of full-length ZIKV/RBV chimeric DNAs

Primer	Polarity	Sequence*	Coordinates†
ZR1-F	Sense	5′ GAATAAGGGCGACACGGAAATGTC 3′	–
ZR1-R	Antisense	5′ CAACAACTCGGTTCACTAAACGAGCTCT 3′	1–8
ZR2-F	Sense	5′ CGTTTAGTGAACCGAGTTGTTGATCTGTGTGAATC 3′	1–21
ZR2-R	Antisense	5′ GCCATCAATGCCATAGCTGTGGTCAGC 3′	455–481
ZR3-F	Sense	5′ CACAGCTATGGCATTGATGGCTATGCAGG 3′	461–489
ZR3-R	Antisense	5′ CCCCACATCTCCCATGACTCCAGTTGTAAATC 3′	2398–2429
ZR4-F	Sense	5′ GTCATGGGAGATGTGGGGTGCTC 3′	2412–2434
ZR4-R	Antisense	5′ GGACGGCTGGGGTAATGAAAGTTGTC 3′	6983–7008
ZR5-F	Sense	5′ GACAACTTTCATTACCCCAGCCGTCC 3′	6983–7008
ZR5-R	Antisense	5′ TGCCGGCCAGACCCATGGATTTC 3′	10,724–10,738
ZR6-F	Sense	5′ TGGGTCTGGCCGGCATGGTC 3′	10,732–10,738
ZR6-R	Antisense	5′ CTCAGGGTCAATGCCAGCGCTTAATTTC 3′	–

*Sequences are colour-coded: cytomegalovirus promoter, black; Zika virus, blue; Rio Bravo virus, red; hepatitis delta virus anti-genomic ribozyme sequence and simian virus 40 polyadenylation signal, green.

†Location of the primer binding sites in the chimeric flavivirus genome with numbers starting at the first nucleotide of the flavivirus 5′ untranslated region (not the first nucleotide of the promoter sequence).

The six overlapping PCR products used to generate the YFV/RBV chimera were designated YR1–YR6 (Fig. 1a). YR1 consisted of 796 bp and encompassed the CVM promoter sequence followed by the first 15 nt of the YFV genome. YR2 (506 bp) encompassed the final 13 bp of the CVM promoter sequence, all of the 5′ UTR and C gene of YFV, and the first 12 nt of the prM gene of RBV. YR3 (1965 bp) encompassed the last 8 nt of the C gene of YFV, all of the prM-E genes of RBV and the first 10 nt of the NS1 gene of YFV. YR4 (4761 bp) encompassed the last 26 nt of the E gene of RBV followed by all of the NS1–NS4A genes and the 5′ half of the NS4B gene of YFV. YR5 (3694 bp) encompassed the 3′ half of the NS4A gene and all of the NS5 gene and 3′ UTR of YFV, followed by the first 8 nt of the HDR. YR6 (202 bp) encompassed the last 10 nt of the 3′ UTR of YFV, followed by the HDR and SV40p.

The six overlapping PCR products used to generate the ZIKV/RBV chimera were designated ZR1–ZR6 (Fig. 1b). ZR1 consisted of 789 bp and encompassed the CVM promoter sequence followed by the first 8 nt of the YFV genome. ZR2 (495 bp) encompassed the final 14 bp of the CVM promoter sequence, all of the 5′ UTR and C gene of YFV, and the first 8 nt of the prM gene of RBV. ZR3 (1969 bp) encompassed the last 13 nt of the C gene of YFV, all of the prM-E genes of RBV and the first 9 nt of the NS1 gene of YFV. ZR4 (4597 bp) encompassed the last 9 nt of the E gene of RBV followed by all of the NS1–NS4A genes and the 5′ half of the NS4B gene of YFV. ZR5 (3764 bp) encompassed the 3′ half of the NS4A gene and all of the NS5 gene and 3′ UTR of YFV, followed by the first 8 nt of the HDR. ZR6 (199 bp) encompassed the last 7 nt of the 3′ UTR of YFV, followed by the HDR and SV40p.

PCR products were purified using the PureLink PCR purification kit (Thermo Fisher Scientific) and then joined using Gibson Assembly Master Mix following the manufacturer’s instructions (New England BioLabs). Briefly, equimolar amounts of the required PCR products (Y1–Y6 or Z1–Z6) were mixed together to give a total amount of 1.67 µg. DNA preparations were diluted in dH_2_0 and added to equal volumes of 2× Gibson Assembly Master Mix such that the final reaction volumes were 20 µl. Reactions were incubated at 50 °C for 3 h.

### Transfections and virus recovery

The entire volume of each Gibson assembly reaction was mixed with 900 µl of serum-free Opti-MEM (Thermo Fisher Scientific) and 12 µl of Lipofectamine 2000 reagent (Thermo Fisher Scientific), incubated for 20 min at room temperature and inoculated onto subconfluent monolayers of BHK-21 cells in 25 cm^2^ flasks. The transfected cells were left overnight and fresh media were added the next day. The cells were incubated for 4–6 days, after which they were frozen and thawed. Two additional passages were performed in BHK-21 or Vero cells. Supernatants were harvested from the final cell culture passages at 5 days post-infection (p.i.) and assayed for virus.

### Reverse-transcription polymerase chain reaction

Total RNA was extracted from supernatants using the QIAamp viral RNA mini kit (Qiagen). Complementary DNAs were generated using Superscript III reverse transcriptase (Thermo Fisher Scientific). PCRs were performed using Phusion high-fidelity DNA polymerase (New England BioLabs) when generating amplicons for chimera construction and *Taq* polymerase (Thermo Fisher Scientific) when assaying cells for the presence of chimeric and parental viruses. RBV, YFV-17D and ZIKV-specific primers were designed using published sequences (GenBank accession no. JQ582840.1, FJ654700.1 and KX377337.1, respectively). All of the primer sequences used in this study are available upon request. PCR products were examined by 1 % agarose gel electrophoresis, purified using QIAquick spin columns (Qiagen) and sequenced using a 3730×1 DNA sequencer (Applied Biosystems).

### Hyperimmune ascitic fluids

Hyperimmune ascitic fluids (HIAFs) from adult mice inoculated with RBV, in addition to heterologous HIAFs from adult mice inoculated with YFV, ZIKV and several other MBFVs, were obtained from the virus reference collections maintained at the University of Texas Medical Branch in Galveston, Texas, USA. Infected brain from suckling mice was used as antigen.

### Immunofluorescence assays

C6/36 and Vero cells were seeded on six-well (9.6 cm^2^) dishes containing 18 mm diameter coverslips at a density of 1×10^5^ cells/well and incubated at 28 and 37 °C, respectively, until they reached confluence. Cells were inoculated with the chimeric or parental viruses at a multiplicity of infection (m.o.i.) of 0.1 or they were mock inoculated and then incubated for 4 days (Vero cells) or 5 days (C6/36 cells). The chimeric viruses had undergone two cell culture passages in Vero cells and, where specified, two additional passages in C6/36 cells, prior to the experiment. Cells were fixed at −20 °C for 3 min with 100 % methanol or at room temperature for 10 min with 2 % paraformaldehyde in phosphate-buffered saline (PBS) and then washed three times with PBS. Fixed cells were permeabilized by incubation with 0.2 % Triton X-100 in PBS for 5 min and then washed three times with PBS. Cells were blocked for 10 min with 2 % bovine serum albumin in PBS and incubated for 1 h with 1/500 HIAFs to RBV or 1/500 heterologous HIAFs to MBFVs. Cells were washed three times with PBS and then incubated for an additional hour with 1/1000 Alexa 594-conjugated rabbit anti-mouse IgG (Thermo Fisher Scientific). Immuno-stained cells were washed three times with PBS and mounted on slides with ProLong reagent with DAPI (4′,6-diamidino-2-phenylindole dihydrochloride) (Thermo Fisher Scientific). Immuno-stained samples were examined with a Zeiss Axiovert 200 inverted microscope equipped with fluorescence optics. Images were prepared using Photoshop and Illustrator software (Adobe Systems).

### Plaque morphology comparisons

Viruses were inoculated onto confluent monolayers of Vero cells in 35 mm culture dishes and incubated at 37 °C for 60 min. Three millilitres of neutral red-deficient minimum essential medium (Thermo Fisher Scientific) supplemented with 2 % FBS, antibiotics and 1 % agar were added to each well and plates were incubated at 37 °C for 5 or 7 days. To fix the cells, 2 ml of 10 % formaldehyde was added directly onto each agar overlay and the plates were incubated at 37 °C for 60 min. Agar overlays were gently removed and 0.5 ml of 0.25 % crystal violet (w/v) in 20 % methanol was added to each well. Once the desired intensity was reached, plates were rinsed several times with tap water and photographed, and then plaque diameters were measured. At least 40 plaques were measured for each virus at each time point.

### Growth curve comparisons

Subconfluent monolayers of C6/36 and Vero cells in 150 cm^2^ flasks were inoculated with the chimeric and parental viruses at an m.o.i. of 0.1. The chimeric viruses had undergone two cell culture passages in Vero cells prior to the experiment. After 1 h, virus inocula were removed and cell monolayers were washed three times with PBS. Supernatants were collected daily for 5 days and stored in aliquots at −80 °C until titrated by plaque assay using Vero cells. Two independent experiments were performed. Within each experiment, six replicates of each virus/dilution/time point were tested. Data were used to calculate mean viral titres±1 standard deviation.

### *In vitro* host range experiments

The *in vitro* host range of ZIKV-RBV(prM-E) was assessed using BTI-Tn-5B1-4, C6/36, CT, DEF, LLC-MK2, murine microglia, Sf9, Sua 4.0 and Vero cell lines. ZIKV-RBV(prM-E) had been passaged twice in Vero cells prior to the experiment. RBV and ZIKV were also included. Briefly, cell monolayers approaching confluence in 25 cm^2^ flasks were inoculated with virus at an m.o.i. of 0.1. The virus inoculum was removed after 1 h and the cell monolayers were washed three times with PBS and incubated in 5 ml of fresh cell culture media for 7 days or until 40–60 % of the cell monolayer displayed CPE. Supernatants were collected and 50 µl of each supernatant was added to 2 ml of cell culture media and inoculated into fresh flasks of cells. After 1 h, the virus inoculum was removed and the cell monolayers were washed and incubated as described above. A total of five passages were performed, with supernatants collected after each passage and tested by RT-PCR for viral RNA. A cell line was considered to support virus replication if RT-PCR products were detected after every passage.

## Results

Two chimeric flaviviruses, designated YFV-RBV(prM-E) and ZIKV-RBV(prM-E), were created by inserting the prM-E genes of RBV into the genetic backbones of YFV and ZIKV, respectively. Each viral chimera was generated from six overlapping PCR products that were joined by Gibson assembly (Fig. 1). Reactions were performed such that the assembled DNAs contained a CMV promoter sequence at their 5′ ends and HDR and SV40pA sequences at their 3′ ends. Assembled DNAs were transfected into BHK-21 cells and then two additional passages were performed in BHK-21 or Vero cells. Supernatants were harvested from the final cell culture passages and tested for virus by plaque assay, revealing successful chimeric virus production.

The complete genomes of the chimeric viruses harvested from the final cell culture passages were sequenced. Seven nucleotide substitutions not present in the corresponding regions of the parental viruses were identified in the genome of YFV-RBV(prM-E) (Table 3). One substitution (coordinate 1134) resulted in a non-synonymous change in residue 53 of the E protein. All other substitutions were synonymous. Eight nucleotide substitutions were identified in the genome of ZIKV-RBV(prM-E). Three mutations (coordinates 1573, 3213 and 8920) resulted in non-conservative changes in residues 202, 265 and 441 of the E, NS1 and NS5 proteins, respectively. Another mutation (coordinate 9631) resulted in a conservative change in residue 678 of the NS5 protein. All other substitutions were silent.

**Table 3.** Mutations accrued in the genomes of YFV-RBV(prM-E) and ZIKV-RBV(prM-E)

Virus	Nucleotide position	Amino acid position	Nucleotide change	Amino acid change
YFV-RBV(prM-E)	142	C-8	G → A	Silent
	1134	E-53	T → C	Leu → Pro
	4030	NS2A-208	T → C	Silent
	6001	NS3-485	T → C	Silent
	6256	NS3-564	G → C	Silent
	9886	NS5-758	C → T	Silent
	10120	NS5-836	G → A	Silent
ZIKV-RBV(prM-E)	1573	E-202	A → C	Asp → Ala
	2411	E-481	A → G	Silent
	3213	NS1-265	A → G	Lys → Glu
	3758	NS2A-94	C → T	Silent
	7394	NS4B-183	C → T	Silent
	8214	NS5-206	T → C	Silent
	8920	NS5-441	A → G	Glu → Gly
	9631	NS5-678	A → G	His → Arg

**Fig. 2.**
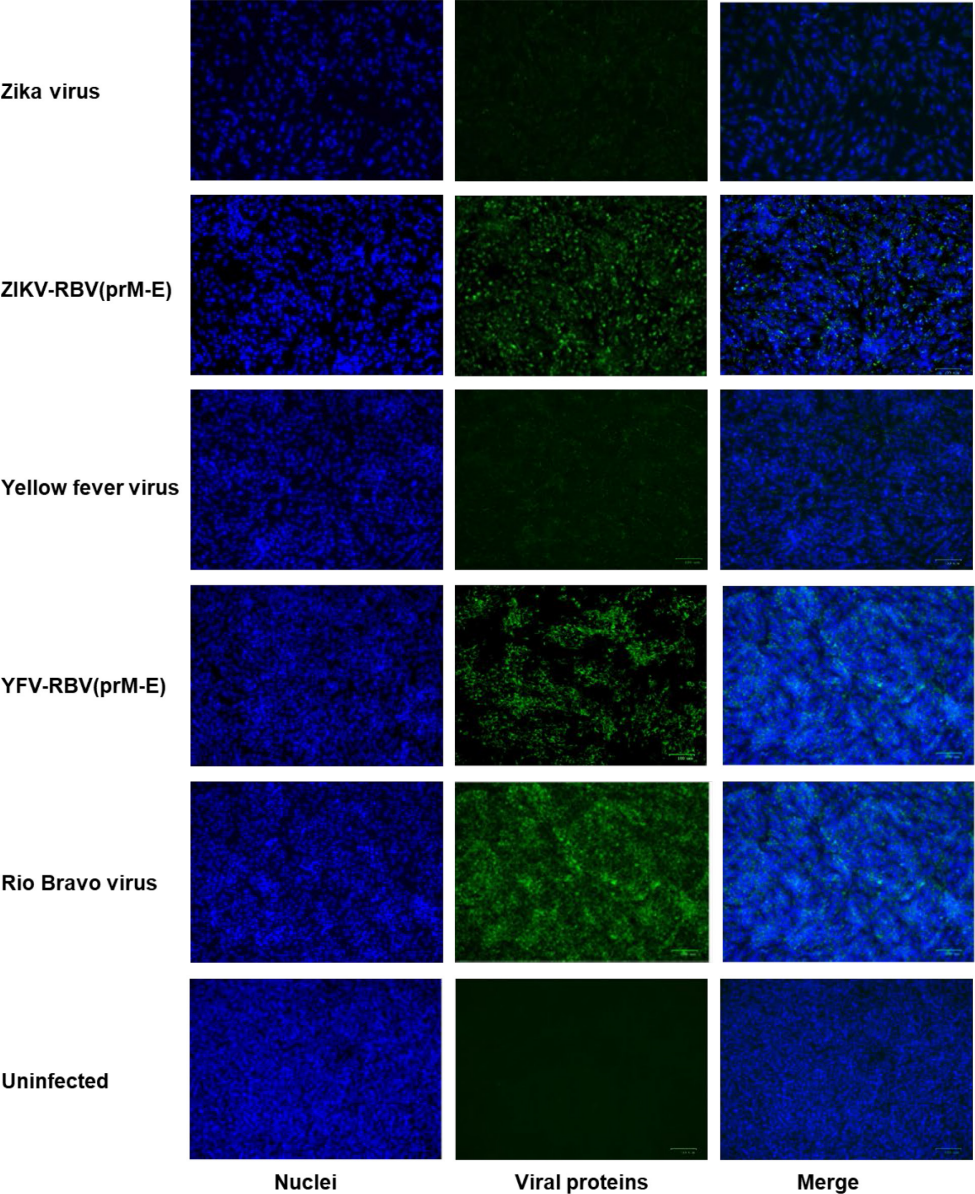
Immunofluorescence analysis of Vero cells inoculated with YFV-RBV(prM-E), ZIKV-RBV(prM-E) and the three parental viruses. Subconfluent monolayers of Vero cells in six-well culture dishes were inoculated with ZIKV, ZIKV-RBV(prM-E), YFV, YFV-RBV(prM-E) or RBV at an m.o.i. of 0.1 (rows 1–5, respectively) or they were inoculated with media only (row 6). The chimeric viruses had undergone two passages in Vero cells prior to the experiment. At 4 days post-inoculation, cells were fixed with methanol and immuno-stained with anti-RBV antibody (column 2), followed by Alexa 594-conjugated donkey anti-rabbit IgG. DAPI was used to visualize the nucleic (column 1). Merged images are also shown (column 3).

**Fig. 3.**
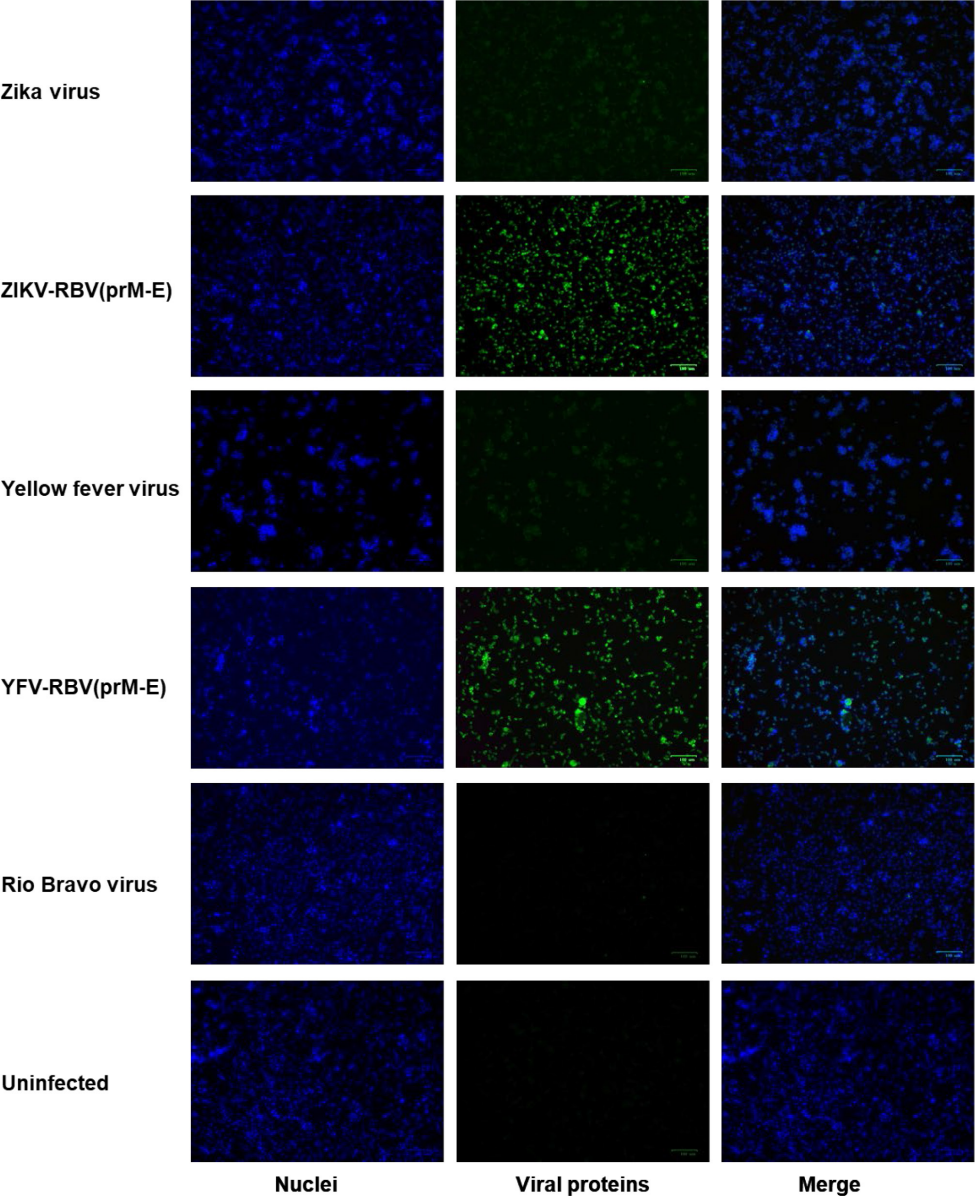
Immunofluorescence analysis of C6/36 cells inoculated with YFV-RBV(prM-E), ZIKV-RBV(prM-E) and the three parental viruses. Subconfluent monolayers of C6/36 cells in six-well culture dishes were inoculated with ZIKV, ZIKV-RBV(prM-E), YFV, YFV-RBV(prM-E) or RBV at an m.o.i. of 0.1 (rows 1–5, respectively) or they were inoculated with media only (row 6). The chimeric viruses had undergone two passages in Vero cells followed by two passages in C6/36 cells prior to the experiment. At 5 days post-inoculation, cells were fixed with methanol and immuno-stained with anti-RBV antibody (column 2), followed by Alexa 594-conjugated donkey anti-rabbit IgG. DAPI was used to visualize the nucleic (column 1). Merged images are also shown (column 3).

**Fig. 4.**
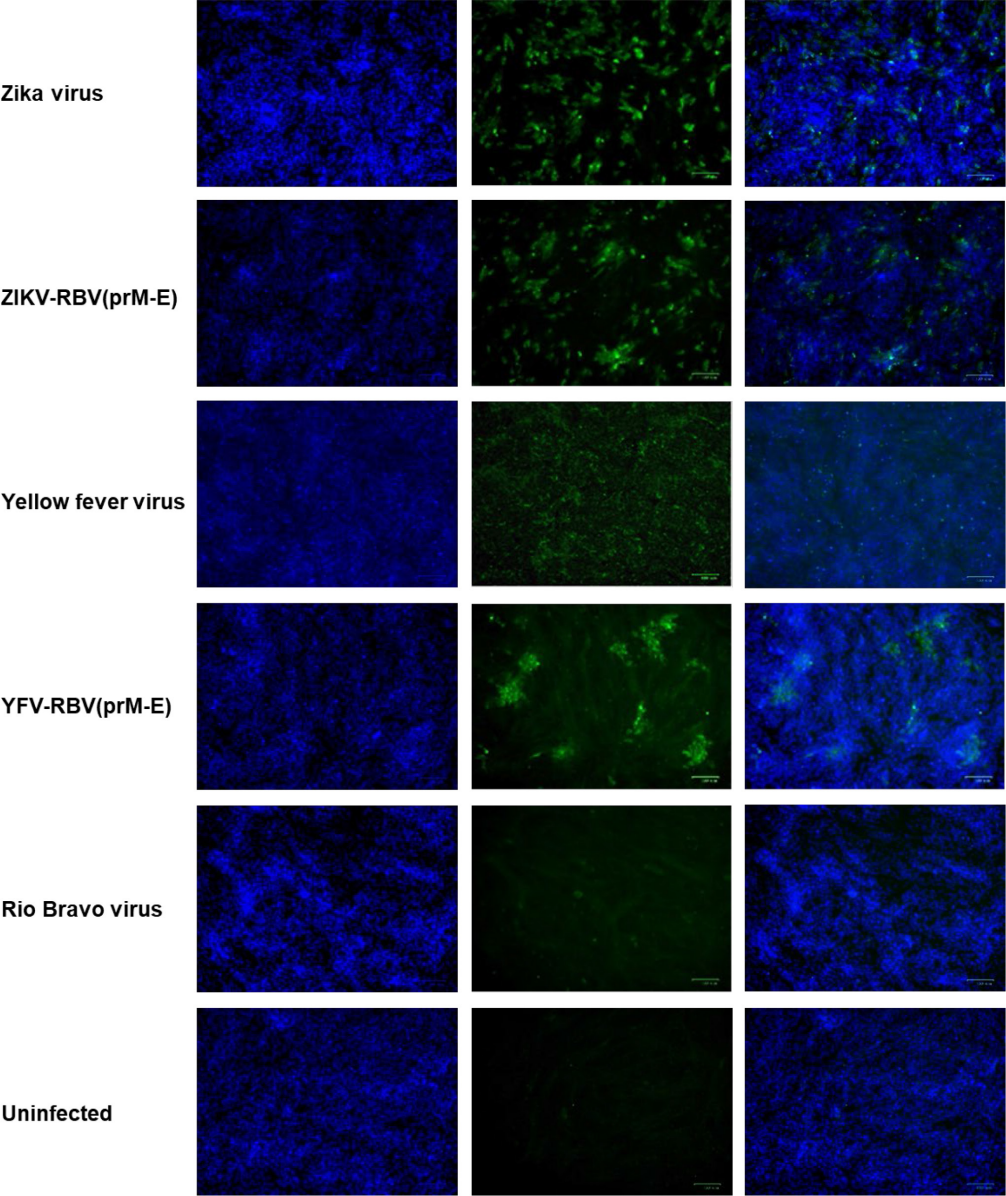
Immunofluorescence analysis of Vero cells inoculated with YFV-RBV(prM-E), ZIKV-RBV(prM-E) and the three parental viruses. Subconfluent monolayers of Vero cells in six-well culture dishes were inoculated with ZIKV, ZIKV-RBV(prM-E), YFV, YFV-RBV(prM-E) or RBV at an m.o.i. of 0.1 (rows 1–5, respectively) or they were inoculated with media only (row 6). The chimeric viruses had undergone two passages in vertebrate cells prior to the experiment. At 4 days post-inoculation, cells were fixed with methanol and immuno-stained with anti-MBFV antibody (column 2), followed by Alexa 594-conjugated donkey anti-rabbit IgG. DAPI was used to visualize the nucleic (column 1). Merged images are also shown (column 3).

**Fig. 5.**
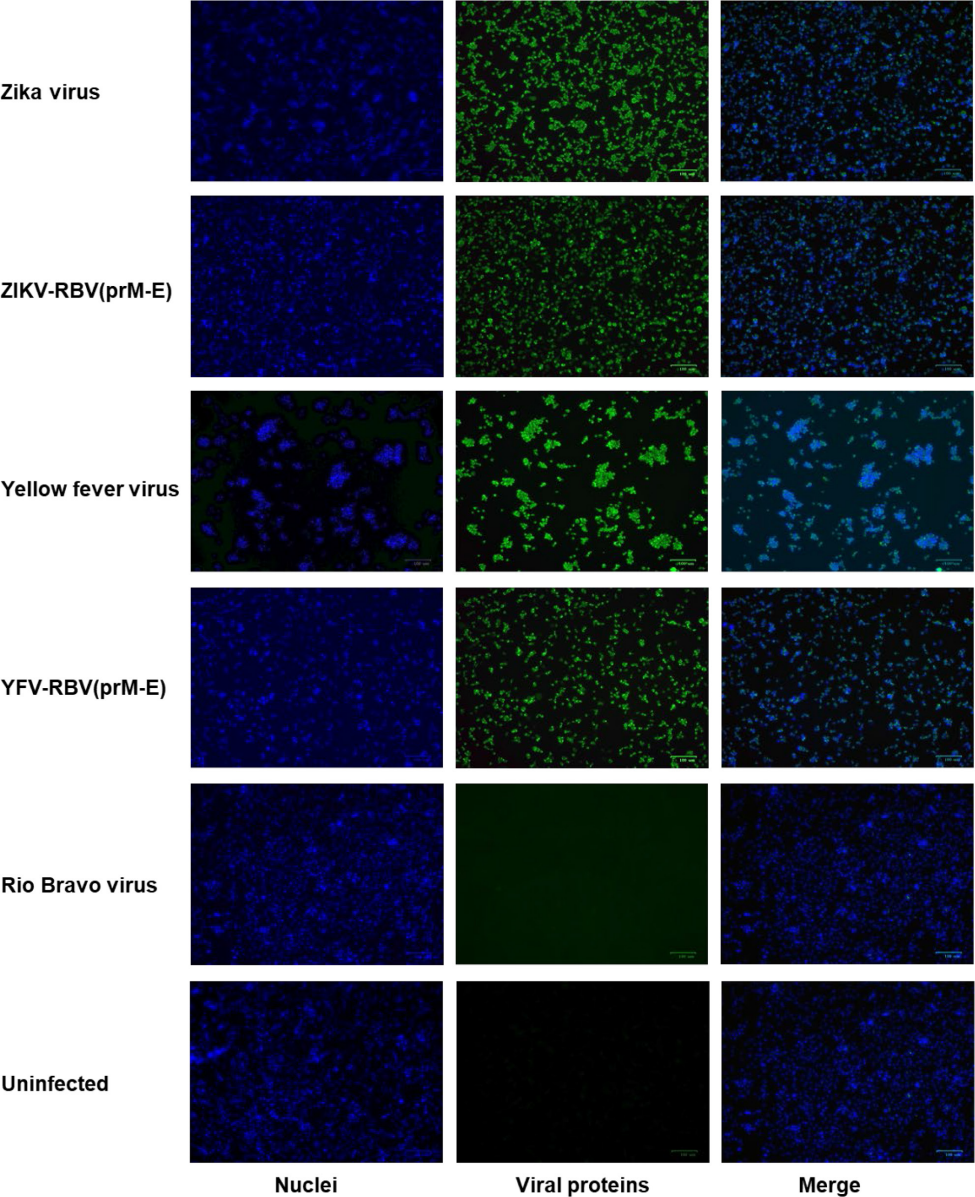
Immunofluorescence analysis of C6/36 cells inoculated with YFV-RBV(prM-E), ZIKV-RBV(prM-E) and the three parental viruses. Subconfluent monolayers of C6/36 cells in six-well culture dishes were inoculated with ZIKV, ZIKV-RBV(prM-E), YFV, YFV-RBV(prM-E) or RBV at an m.o.i. of 0.1 (rows 1–5, respectively) or they were inoculated with media only (row 6). The chimeric viruses had undergone two passages in Vero cells followed by two passages in C6/36 cells prior to the experiment. At 5 days post-inoculation, cells were fixed with methanol and immuno-stained with anti-MBFV antibody (column 2), followed by Alexa 594-conjugated donkey anti-rabbit IgG. DAPI was used to visualize the nucleic (column 1). Merged images are also shown (column 3).

Additional evidence that YFV-RBV(prM-E) and ZIKV-RBV(prM-E) replicate in C6/36 and Vero cells was provided by immunofluorescence assay (Figs 2–5). HIAFs to RBV detected antigen in Vero cells inoculated with ZIKV-RBV(prM-E) and YFV-RBV(prM-E) (Fig. 2). The HIAFs to RBV also detected antigen in C6/36 cells inoculated with the chimeric viruses (Fig. 3). As expected, the HIAFs detected antigen in Vero, but not C6/36, cells inoculated with RBV. Antigen was undetectable in all YFV-, ZIKV- and mock-inoculated cell cultures. When the HIAFs to MBFVs were used, antigen was detected in Vero cells inoculated with YFV and ZIKV (Fig. 4). The HIAFs failed to detect antigen in Vero cells inoculated with RBV, suggesting that the anti-MBFV prM and E antibodies in the HIAFs do not cross-react with RBV antigen. Interestingly, the HIAFs to MBFVs detected antigen in Vero cells inoculated with the chimeric viruses. We attributed this finding to the presence of anti-MBFV NS1 antibodies in the HIAFs. The HIAFs to MBFVs also detected antigen in C6/36 cells inoculated with the two chimeric viruses (Fig. 5).

**Fig. 6.**
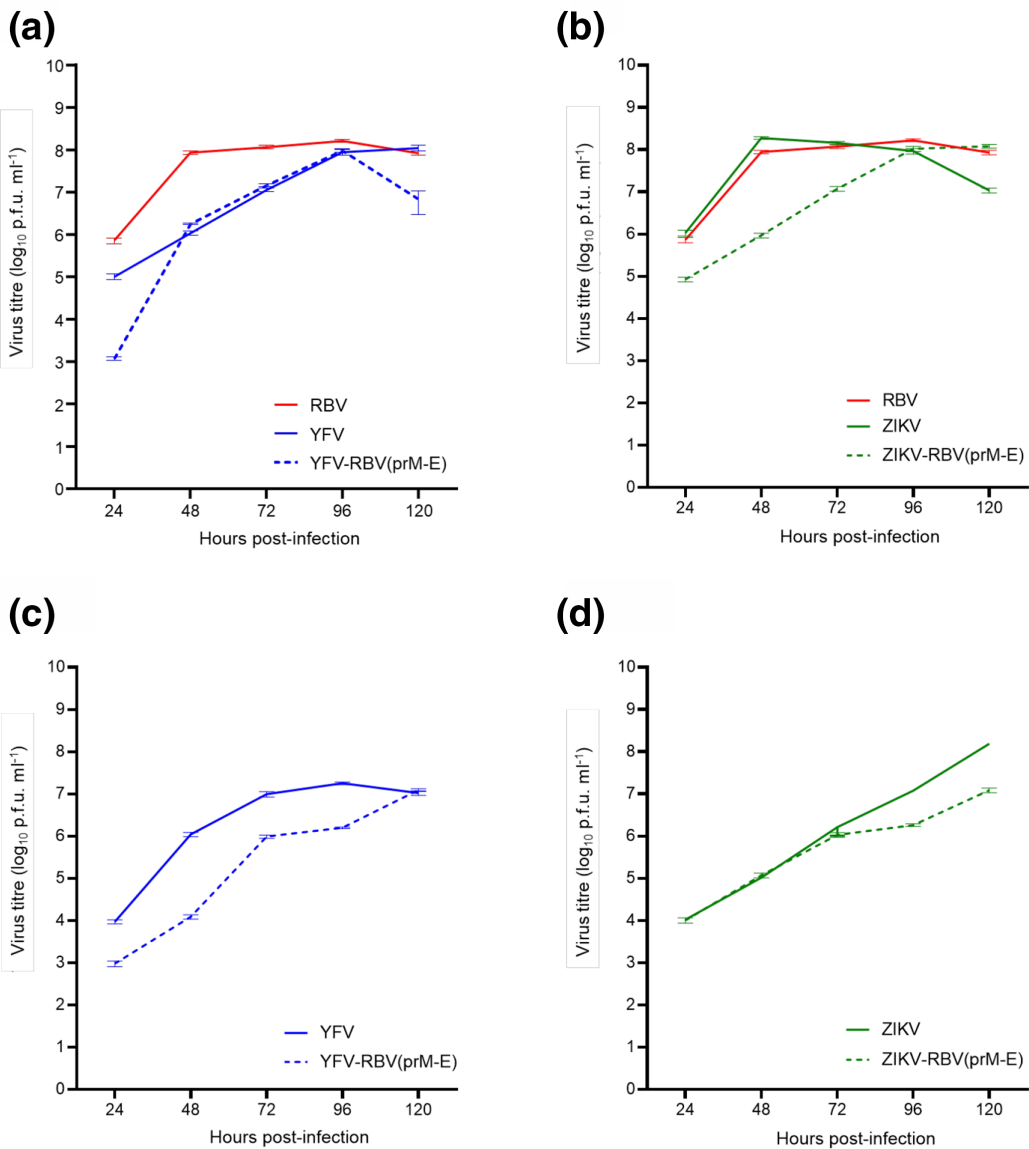
Comparison of the *in vitro* replication kinetics of the chimeric and parental viruses in Vero and C6/36 cells. Subconfluent monolayers of (**a, b**) Vero cells and (**c, d**) C6/36 cells were inoculated with (**a, c**) YFV-RBV(prM-E), RBV and YFV or (**b, d**) ZIKV-RBV(prM-E), RBV and ZIKV at an m.o.i. of 0.1. Supernatants were collected daily for 5 days and tested by plaque assay. The chimeric viruses had undergone two passages in Vero cells prior to the experiment.

The growth and yields of the chimeric and parental viruses were measured in Vero and C6/36 cells at 24, 48, 72, 96 and 120 h p.i. (Fig. 6). In Vero cells, all viruses produced similar mean peak titres, but the chimeric viruses grew more slowly than their parental viruses at earlier time points. YFV-RBV(prM-E) and ZIKV-RBV(prM-E) reached mean peak titres of 8.0 (±0.03) log_10_ p.f.u. ml^−1^ at 96 h p.i. and 8.1 (±0.07) log_10_ p.f.u. ml^−1^ at 120 h p.i., respectively. RBV, YFV and ZIKV reached mean peak titres of 8.2 (±0.03) log_10_ p.f.u. ml^−1^ at 96 h p.i., 8.1 (±0.08) log_10_ p.f.u. ml^−1^ at 120 h p.i. and 8.3 (±0.03) log_10_ p.f.u. ml^−1^ at 48 h p.i. In C6/36 cells, YFV-RBV(prM-E) replicated more slowly than YFV at earlier time points, but the two viruses produced similar mean peak titres. YFV-RBV(prM-E) reached a mean peak titre of 7.1 (±0.03) log_10_ p.f.u. ml^−1^ at 120 h p.i. and YFV reached a mean peak titre of 7.3 (±0.03) log_10_ p.f.u. ml^−1^ at 96 h p.i. ZIKV-RBV(prM-E) replicated more slowly than ZIKV at later time points and its mean peak titre was approximately 10-fold lower. ZIKV reached a mean peak titre of 8.2 (±0.12) log_10_ p.f.u. ml^−1^ at 120 h p.i. and ZIKV-RBV(prM-E) reached a mean peak titre of 7.1 (±0.05) log_10_ p.f.u. ml^−1^ at 120 h p.i.

**Fig. 7.**
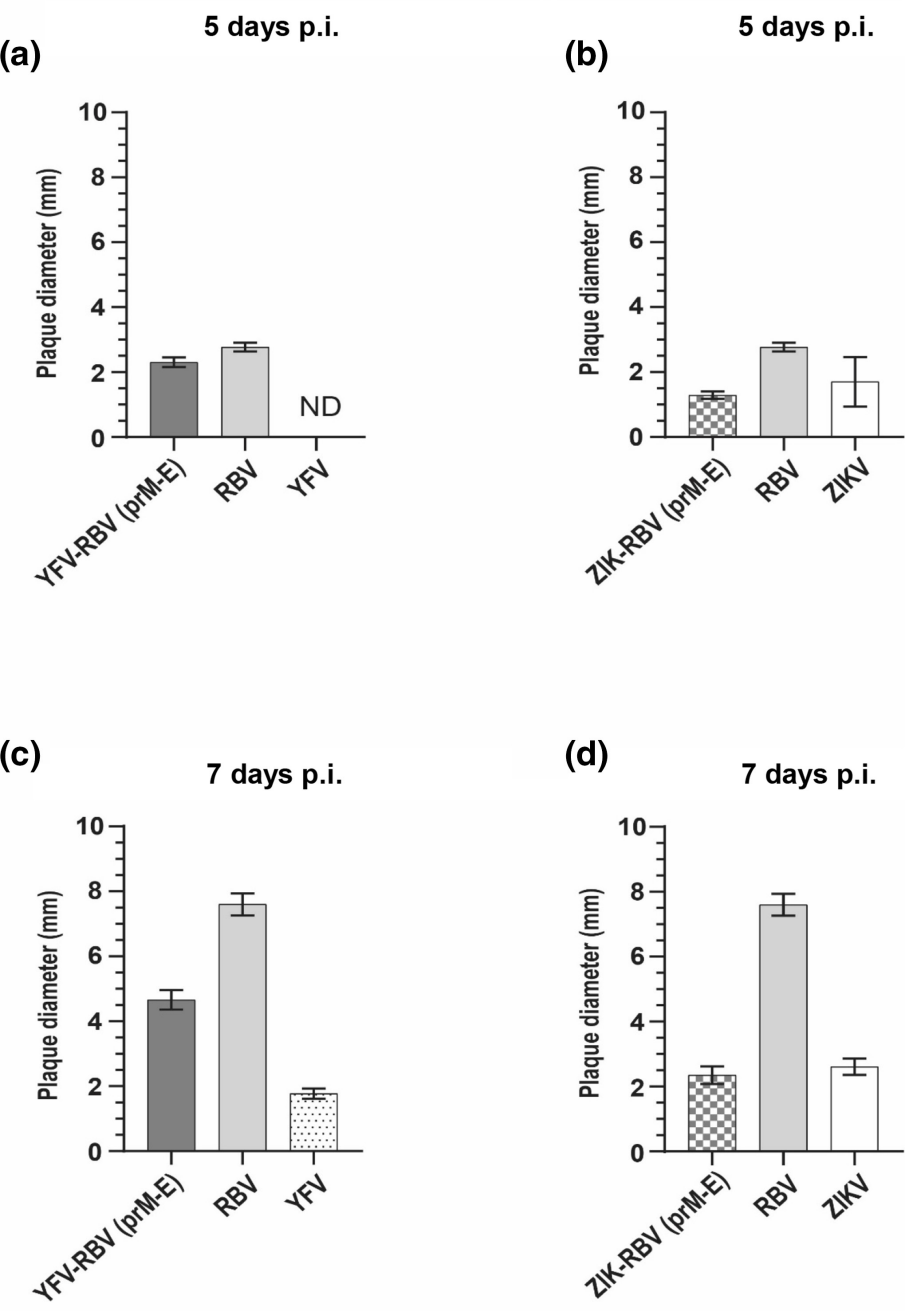
Comparison of the mean diameters of plaques produced by the chimeric viruses and their parental viruses in Vero cells at 5 and 7 days post-infection. Confluent monolayers of Vero cells were inoculated with (**a, c**) YFV-RBV(prM-E) and its parental viruses or (**b, d**) ZIKV-RBV(prM-E) and its parental viruses. Cells were inoculated for (**a, b**) 5 days or (**c, d**) 7 days and fixed. At least 40 plaques were measured for each virus at each time point, with data expressed as mean plaque diameters±1 standard deviation. nd, not detected.

YFV-RBV(prM-E) and ZIKV-RBV(prM-E) exhibited differential plaque phenotypes from their respective parental viruses in Vero cells (Fig. 7). YFV-RBV(prM-E) produced an intermediate plaque phenotype, forming plaques that were larger than YFV plaques but smaller than RBV plaques. ZIKV-RBV(prM-E) plaques were smaller than those of both of its parental viruses. At 5 days p.i., YFV-RBV(prM-E) and ZIKV-RBV(prM-E) plaques had mean diameters±1 standard deviation of 2.30±0.15 and 1.29±0.11 mm, respectively. RBV and ZIKV plaques were 2.77±0.13 and 1.70±0.76 mm, respectively, and YFV plaques were too small (<0.1 mm) to measure accurately. At 7 days p.i., YFV-RBV(prM-E) and ZIKV-RBV(prM-E) plaques were 4.66±0.30 and 2.35±0.27, respectively. RBV, YFV and ZIKV plaques were 7.60±0.34, 1.77±0.16 and 2.61±0.25, respectively. The analysis of variance (ANOVA) F-test showed a significant difference among the plaque sizes of YFV-RBV(prM-E), YFV and RBV on day 5 (F=108, DF=2, 43, *P*-value <0.0001) and day 7 (F=140.3, DF=2, 45, *P*-value <0.0001). The ANOVA F-test also showed a significant difference among the plaque sizes of ZIKV-RBV(prM-E), ZIKV and RBV on both day 5 (F=37.2, DF=2, 84, *P*-value <0.0001) and day 7 (F=74.4, DF=2, 34, *P*-value <0.0001). All pairwise comparisons were significant (*P*<0.05) using Tukey’s adjustment, except for YFV-RBV(prM-E) and YFV on day 5 (*P*=0.0613) and ZIKV-RBV(prM-E) and ZIKV on day 7 (*P*=0.7550).

The *in vitro* host range of ZIKV-RBV(prM-E), along with its two parental viruses, was further assessed using seven additional cell lines: *An. gambiae* (Sua 4.0), *Cx. tarsalis* (CT), duck embryonic fibroblast (DEF), murine microglia, rhesus macaque (LLC-MK2), *S. frugiperda* (Sf9) and *T. ni* (BTI-Tn-5B1-4) cell lines (Table 4). C6/36 and Vero cells were also included. A cell line was considered to support virus replication if viral RNA was detected by RT-PCR after all five passages. Cell monolayers were washed three times after the inoculum was removed to ensure that non-replicating viral RNA was not present. All three viruses replicated in DEF, LLC-MK2 and Vero cells, but not murine microglia cells. Additionally, replication of ZIKV-RBV(prM-E) and ZIKV occurred in C6/36 cells, but none of the other invertebrate cell lines. RBV failed to replicate in all invertebrate cell lines.

**Table 4.** Comparison of the *in vitro* host ranges of ZIKV-RBV(prM-E) and its parental viruses

Cell line	Virus		
	Rio Bravo virus	Zika virus	ZIKV-RBV(prM-E)
*Mosquito cells*			
C6/36	−	+	+
CT	−	−	−
Sua 4.0	−	−	−
*Lepidopteran cells*			
BTI-Tn-5B1-4	−	−	−
Sf9	−	−	−
*Vertebrate cells*			
DEF	+	+	+
LLC-MK2	+	+	+
Murine microglia	−	−	−
Vero	+	+	+

The chimeric virus had been passaged twice in Vero cells prior to the experiment. The first passage cultures were inoculated with virus at an m.o.i. of 0.1. A total of five passages were performed. A cell line was considered to support virus replication if RT-PCR products were detected after every passage.

## Discussion

Limited research has been performed on NKV flaviviruses compared to MBFVs, largely because they are not commonly associated with human or animal disease. Four cases of human disease have or are suspected to have occurred because of NKV flavivirus infections acquired outside of the laboratory [26–29]. Among these is a case of RBV. Seven laboratory-acquired cases of RBV have also occurred, with symptoms ranging from mild febrile illness to central nervous system involvement [26, 30, 31]. Although NKV flaviviruses are not considered to be major pathogens, it is important that they are studied. Comparative studies on NKV and MBFVs could provide unique insights into why MBFVs cycle between disparate (vertebrate and mosquito) hosts, often causing serious disease, while NKV flaviviruses do not. Here, we characterized chimeric MBFVs that contain the surface glycoprotein genes of a NKV flavivirus from the bat-associated group and provide evidence that the host restriction of B-NKV flaviviruses within invertebrate cells occurs post-entry.

Five chimeric viruses of MBFVs and rodent-associated NKV (R-NKV) flaviviruses have also been created and characterized to provide an insight into the genetic determinants that condition the host restriction of R-NKV flaviviruses [32–35]. Two chimeric viruses contain the prM-E genes of MODV in the genetic backgrounds of YFV and dengue virus 2, one chimeric virus contains the prM-E genes of West Nile virus in the genetic background of MODV and two chimeric viruses contain select regions of the 3′ UTR of MODV in the genetic background of dengue virus 4. MODV is an R-NKV flavivirus of deer mice [7, 36, 37]. The chimeric viruses containing the prM-E genes of MODV in the genetic backgrounds of YFV and dengue virus 2 replicated in mosquito cells, suggesting that the host restriction of R-NKV flaviviruses occurs post-entry and is not mediated by the viral structural glycoproteins. Taken together, our data and the findings from the aforementioned studies indicate that the host restriction of NKV flaviviruses belonging to both the bat- and rodent-associated groups does not occur at the level of attachment or entry.

Insect-specific flaviviruses (ISFs) are another group of flaviviruses with restricted host ranges [6, 10, 38]. ISFs can be divided into classical ISFs (cISFs) and dual host-affiliated ISFs (dISFs). Classical ISFs were the first to be discovered and are phylogenetically distinct from all other flaviviruses, whereas dISFs phylogenetically affiliate with MBFVs, despite their apparent vertebrate-restricted phenotype. Chimeric viruses of ISFs and MBFVs have been created and characterized in order to identify the genetic determinants that condition the ISF host range restriction [39–46]. Chimeric MBFVs containing the prM-E genes of dISFs could not replicate in vertebrate cells, although viral antigen was produced [43]. Chimeric MBFVs containing the prM-E genes of cISFs were also incapable of replicating in vertebrate cells [39–42]. However, this replication-deficient phenotype was overcome when zinc-finger antiviral protein (ZAP) knockout vertebrate cells cultured at 34 °C were used [46]. The ZAP in vertebrate cells specifically binds to CpG dinucleotides in single-stranded viral RNAs, reducing viral replication [47]. ISFs contain more CpG dinucleotides in their genomes compared to MBFVs [6]. Because chimeric MBFVs containing the prM-E genes of cISFs and dISFs cannot replicate in wild-type vertebrate cells under standard temperature conditions, while those containing the prM-E genes of B-NKV and R-NKV flaviviruses can replicate in mosquito cells, different mechanisms may condition the host range restrictions of vertebrate- and mosquito-specific flaviviruses.

Both chimeric viruses replicated more slowly than their respective parental viruses in vertebrate cells, but eventually produced similar peak titres. The replication kinetics of YFV-RBV(prM-E) was more similar to YFV than to RBV. Other studies have also shown that chimeric flaviviruses created by prM-E gene substitutions exhibit replication kinetics that are closer to the parental virus that contributed the nonstructural genes [32, 48–51]. In contrast, the replication kinetics of ZIKV-RBV(prM-E) did not resemble one parental virus noticeably more than the other. Although both chimeric viruses replicated more slowly than their respective parental viruses in vertebrate cells, the difference was more pronounced with ZIKV-RBV(prM-E). This could indicate that the genetic exchange between ZIKV and RBV was not as well tolerated as that between YFV and RBV. In this regard, ZIKV-RBV(prM-E) exhibited a reduced plaque size compared to its parental viruses, whereas YFV-RBV(prM-E) produced an intermediate plaque phenotype. If the genetic exchange between ZIKV and RBV was less tolerated, it could explain why the peak titre of ZIKV-RBV(prM-E) was approximately 10-fold lower than ZIKV in mosquito cells, while the YFV-RBV(prM-E) and YFV produced similar peak titres. However, the titres of three viruses, ZIKV-RBV(prM-E), ZIKV and YFV, were not in decline at the final time point and therefore higher titres may have been reached if the experiments had continued beyond 120 h p.i. The genomes of YFV-RBV(prM-E) and ZIKV-RBV(prM-E) contained one and four nonsynonymous mutations, respectively, and the effects of the mutations on the *in vitro* host ranges, replication kinetics and plaque morphologies of the chimeric viruses are not known. The single nonsynonymous mutation in the genome of YFV-RBV(prM-E) and one of the nonsynonymous mutations in the genome of ZIKV-RBV(prM-E) were located in the E region at E-53 (Leu → Pro) and E-202 (Asp → Ala), respectively. We cannot dismiss the possibility that these mutations permitted entry into mosquito cells and that entry would not have otherwise occurred. This is especially true for YFV-RBV(prM-E) because the mutation at E-53 is a reversion to YFV sequence. However, the mutation at E-202 is not a reversion to the ZIKV sequence. ZIKV contains an Asn at this site.

Consistent with earlier reports, we demonstrated that RBV replicates in DEF, LLC-MK2 and Vero cells, but not C6/36 and CT cells [14, 20–22, 26]. We provide additional evidence that RBV lacks the ability to replicate in arthropod cells by revealing that it cannot replicate in *Anopheles* and lepidopteran cell cultures. The *in vitro* host range of ZIKV-RBV(prM-E) was the same as that of ZIKV but distinct from the virus contributing the prM-E genes, further suggesting that the structural glycoproteins of B-NKV flaviviruses are not the genetic elements responsible for their host range restriction.

To conclude, we constructed chimeric MBFVs that contain the prM-E genes of RBV and revealed that they possess the ability to replicate in mosquito cells, providing evidence that the host range restriction of B-NKV flaviviruses does not occur at attachment or entry. The prM-E genes of flaviviruses from each single-host group (B-NKV flaviviruses, R-NKV flaviviruses, cISFs and dISFs) have now been inserted into the genetic backgrounds of MBFVs and the subsequent characterization of the resulting chimeric viruses has provided a useful insight into the genetic determinants that condition the differential host ranges of flaviviruses.
